# Chronic patient as intermittent partner for policy-makers: the case of patient participation in the fight against diabetes and HIV/AIDS in Mali

**DOI:** 10.1186/s12889-019-7453-2

**Published:** 2019-08-28

**Authors:** Jessica Martini, Annick Tijou Traoré, Céline Mahieu

**Affiliations:** 1 Université libre de Bruxelles, School of Public Health, Route de Lennik 808, Brussels, Belgium, CP 596, 1070 Brussels, Belgium; 2 Research laboratory LAM (Les Afriques dans le Monde) / Institute of Political Studies, CNRS/UMR 5115/University of Bordeaux, 11 allée Ausone, Domaine universitaire, 33607 Pessac, Bordeaux France; 3 Université libre de Bruxelles, School of Public Health, Route de Lennik 808, Brussels, Belgium, CP 596, 1070 Brussels, Belgium

**Keywords:** Health policy, Participation, Patient associations, Non-communicable diseases, Diabetes, HIV/AIDS, Chronic diseases, Overseas development assistance, Developing countries, Mali

## Abstract

**Background:**

National and international strategies have increasingly promoted chronic patient participation at different levels of the health care system, building the image of an ‘active’ chronic patient engaged for example in his/her daily self-care and within associations dealing with service delivery and/or policy advocacy. Drawing upon examples of the fight against diabetes and HIV/AIDS in Mali, this article explores the factors that influence the engagement of patient associations at policy level. We focus on the openness of the institutionalised political system, and explore the role that public authorities, caregivers and donors give to diabetes and HIV/AIDS patients.

**Methods:**

Data was collected between 2008 and 2014 in Bamako in the framework of a qualitative research. Thirty-eight actors fighting against diabetes were interviewed, as well as 17 representatives of donors. For HIV/AIDS, 27 actors were interviewed. In both cases, non-participant observation was carried out and documentary sources were collected. Based on theory of public and collective action, a historical and cognitive approach was adopted. Data analysis followed an inductive and iterative method.

**Results:**

Partnerships between public authorities and diabetes patient associations have been intermittent over time and remained rather informal. In the case of people living with HIV/AIDS, the partnership between their associations and public authorities has steadily grown and was progressively institutionalised. Three political factors explain this difference: focus and extent of the commitment of public authorities, existing policy-making processes, and how the law frames patients’ roles. Moreover, opportunities for patient participation depend on the nature and extent of the support provided by international donors. Finally, the cognitive dimension is also at stake, notably in relation to the way the two diseases and patients have been perceived by public authorities, caregivers, and donors.

**Conclusions:**

Chronic patients are *intermittent partners* for policy-makers. Despite the image of *chronic active patients* conveyed by national and international public health strategies, patient participation is not straightforward. Rather, political, economic, and cognitive factors underpin the presence of political opportunities that enable patient participation. Chronicity of the disease appears to play an ambiguous role in the shaping of these factors.

**Electronic supplementary material:**

The online version of this article (10.1186/s12889-019-7453-2) contains supplementary material, which is available to authorized users.

## Background

Sociological literature on chronic diseases shows how living with long-term illness lets patients acquire experiential knowledge that they can use to supplement and challenge the scientific knowledge of healthcare professional [1]. Recognising this lay expertise, national and international strategies have increasingly promoted chronic patient participation, building the image of an ‘active’ chronic patient engaged at different levels of the health care system [2, 3]. Patient participation occurs de facto primarily at the micro- (individual) level, through dialogue with caregivers or in daily self-care [4]. It can also be collective, within patient associations engaged in peer education and service delivery at the meso-level (the one of community and health care organisation) [5], or through representation and advocacy for patients’ rights and interests at the macro- (policy) level [6, 7].

Drawing upon examples of the fight against diabetes and HIV/AIDS in Mali, this article explores the factors that influence patients’ collective engagement at the national policy level. Factors related to how patients themselves build their movements, more specifically, their rationale for engaging within associations and the place they (pro) actively take at policy level have been explored elsewhere [8]. We focus instead on factors that shape what literature on social movements defines as ‘political opportunity structure’, which refers to “aspects of the political system that affect the possibilities that challenging groups have to mobilise effectively” [9]. This notion is related to several aspects [10]. We focus on the openness of the Malian institutionalised political system, and explore the role that public authorities, caregivers and donors give to diabetes and HIV/AIDS patients. Understanding these factors is crucial to develop effective participatory strategies for prevention and control of chronic diseases [11]. It ensures that policy makers acknowledge the complexity of participation dynamics and go beyond the overly simplistic view of a linear relationship between chronicity of a disease, patients’ acquired expertise on a disease, and patients’ capacity to organise and participate in health decision-making.

### Goals, forms and dynamics of patient participation

Within the health sector, strategies encouraging greater participation of citizens in public policy date as far back as the Alma-Ata declaration and the Ottawa charter for health promotion, which acknowledged participation as an individual’s right and duty and considered individuals as the main resource for health respectively [12, 13]. These strategies respond both to claims made by citizens about their right to participate [14] and to government and caregivers’ expectations of improving the effectiveness of healthcare [3]. It is along these lines that Litva et al. identify five types of arguments to justify public participation: instrumentalist arguments aiming to promote or defend participants’ interests; communitarian arguments focused on the interests of particular groups; educative arguments concerned with citizen empowerment; expressive arguments related to political identity; and the wish to improve local accountability [15].

Based on these multiple goals, democratisation of the health sector has taken several forms whereby patient participation holds different meanings for different actors in different contexts [16]. Participation is grounded in various concepts such as public engagement, shared-decision making, or accountable health care [17], and ranges from patients’ information and consultation to their inclusion in joint partnerships [18]. These participatory mechanisms are implemented internationally, most often in high-income countries, but also in middle- and low-income ones, where the norms and values of democracy have been progressively integrated into the health systems [19, 20]. This democratisation is in part due to local health movements, such as in Brazil [21], or, for the most part, owing to funding received by international donors [22].

As stated above, patient participation results from the interrelationship between several factors that mirror complex social dynamics. Theory of public and collective action emphasises the autonomy and capacity of actors to engage and make strategic choices, albeit within the constraints linked to their specific context [23]. How actors engage in practice - their forms of engagement and the partnerships they establish – indeed depends on the resources at their disposal [24, 25], as well as on shared frameworks of understanding in relation to problems, solutions, and the rationale to engage [26]. These elements are intertwined with the political opportunity structure in place. As previously mentioned, this structure strongly depends on the context and may change over time [9]. In terms of patient participation, which is our main focus, experiential knowledge alone is not enough to support patients’ capacity to engage effectively, whether individually or collectively. This is especially true at the policy level where specific competences (negotiation, advocacy, etc.) are needed and power relations are at stake [27]. Particularly influential are the views and strategies of actors (other than patients) who participate in policy processes, such as policy-makers and caregivers. Equally important are international donors for low-income countries that depend on external funding to set and implement policies, and where donors figure amongst the key actors in shaping public action [28].

### The cases of diabetes and HIV/AIDS

We chose to study patient participation whilst comparing the case of diabetes and HIV/AIDS because the similarities and differences between the two diseases make it easier to identify factors associated with patient participation in policy processes.

Both diabetes and HIV/AIDS share a chronic nature, but the experience of chronicity is more recent for people living with HIV/AIDS (PLWHA), as it has followed the discovery of antiretroviral therapy and its affordability in low-income countries. This invites greater scrutiny of the very impact of chronicity on patients’ engagement. Moreover, diabetes is non-communicable and HIV/AIDS is communicable, which may also induce differences in the way actors perceive the diseases and engage with patients.

If we compare the histories of the two diseases, differences can also be noted in the political, social, and scientific contexts within which actors have involved themselves. The manner in which patients have engaged at the global scale also differs. Global mobilisation around diabetes (and other non-communicable diseases, NCDs) still appears very weak at present [29]. In stark contrast, HIV/AIDS has been emblematic of the emergence of the *“reformer patient”* [30], informed about his/her illness, responsible for his/her own care and for health promotion, whilst being an advocate of patients’ rights. Understanding the links between context and patient participation is therefore key to studying participative dynamics.

Ultimately, the involvement of patients affected by diabetes has been studied at the micro- and meso-levels in North America and Europe, whereas their policy participation has received little attention [31]. In comparison, extensive literature exists on PLWHA mobilisation at all three levels and in different contexts [32–34].

### The context of Mali

Diabetes was already present in the 1970s in Mali [35], and the first case of AIDS was officially diagnosed in 1985 [36]. Since then, the country has been classified as “low-prevalence” for both diseases: recent estimates stand at about 1.8% for diabetes [37] and 1% for HIV/AIDS [38]. Despite these low prevalence rates, Mali is amongst the few African countries that boasts of a community centre for diabetic care since the 1990s. Moreover, the country’s community centre for HIV/AIDS treatment is often depicted as a model in the region [39].

With regards to the health sector, specific initiatives have supported citizens’ participation. The Bamako Initiative, launched internationally in 1987 [40] and implemented in Mali as well, has promoted the co-financing and co-management of public health services. Since the 1990s, the implementation of a health sector-wide approach included coordination and participatory mechanisms for all stakeholders [41], starting from civil society organisations to donors. However, ranked amongst the least developed countries [42], Mali is highly dependent on international support, with 45% of its 2011 health budget being funded by donors [43]. This raises an important issue of determining the extent to which situations observed locally reveal ownership of strategies defined internationally and reinterpreted locally [44].

To our knowledge, this is the first study held in Mali that adopts a comparative perspective and explores diabetes and HIV/AIDS patients’ participation at the policy level. Studies done to date in Mali address each disease separately, and focus either on bio-medical [45, 46], anthropological [36, 47, 48], or developmental issues [49, 50].

## Methods

This article is the result of a qualitative research conducted in Bamako, the capital city of Mali. Four field missions were held in 2008 (12 weeks), 2010 (3 weeks), 2012 (2 weeks), and in 2014 (1 week) respectively. Data was collected by means of semi-structured interviews, non-participant observation, and document collection. Multiple methods were used to decrease the limits and biases of each method and improve findings, analysis, and interpretation accordingly [51].

### Participants/study subjects

The four missions were dedicated to the study of public action around diabetes and enabled us to interview 55 actors.^1^ Amongst them, 38 actors were active in the fight against diabetes, including caregivers working both at hospital and community facilities and involved in local associations (5); representatives^2^ of diabetes patient associations (8); representatives of the Malian public administration (9); as well as representatives of local and international non-governmental organisations (NGO, 16). We also interviewed 17 representatives of bilateral and multilateral donors, first targeting donors supporting the health sector in general, and then donors funding diabetes more specifically [see Additional file 1]. The missions held in 2010 and 2014 were also dedicated to data collection pertaining to the fight against HIV/AIDS. All in all, 23 actors were interviewed,^3^ including caregivers engaged in community facilities (3), patient representatives (4), donors (4), representatives of public administration (6), as well as representatives of local and international NGOs (10) [see Additional file 2].

### Sampling and interview process

Interviewees were selected following a purposive sampling approach aiming to include the main stakeholders involved in public action around diabetes and HIV/AIDS. An initial list of stakeholders was drawn up based on internet searches and key informants. The list was then completed through a snowball sampling technique, whereby interviewees were asked about other key stakeholders. Participants were contacted either by phone or through visits at their organisation headquarters. All interviews were conducted in French and face-to-face by the first author. Most interviews were conducted individually and the rest in groups of two or three people depending on their availability. Verbal consent to participate was always acquired prior to the interviews and the scope of our research was clearly stated. Most of the interviews were tape-recorded, upon agreement by the interviewees; hand-written notes were also taken. Interviews lasted approximatively 30 min to 1.5 h. The interview grid was adapted to each stakeholder and aimed to explore the main aspects concerning their engagement in public action around diabetes and/or HIV/AIDS (context, resources, perceptions, interests, internal organisation and partnerships, as well as changes observed over time). In 2014, wrap-up meetings were also organised with local partners of our research programs, including caregivers, patient associations and local NGO involved in the fight against diabetes and HIV/AIDS. The meetings aimed to present our research findings to the local stakeholders; discussion with them enabled us to validate the study results and to complete or adjust them based on the new information provided during the meeting.

### Observation

Field missions were also the occasion to conduct specific non-participant observation. The first author participated to trainings on diabetes care, visited a diabetes unit within a municipal health facility, and attended a closing ceremony of World Diabetes Day held by a patient association [see Additional file 3]. Two specific non-participant observations were also carried out for HIV/AIDS: the first was in the framework of an awareness event organised by a community health-care facility, and the second during a meeting between stakeholders funding HIV/AIDS, local authorities, and civil society [see Additional file 4]. Observations followed a convenience sampling based on invitations offered by the interviewees and meetings taking place during our field mission. The purpose was to collect primary data on activities held about diabetes and HIV/AIDS, to better understand how the two diseases are framed locally and to identify key stakeholders and priorities. Written notes about speeches and key aspects of the meetings observed were taken in field notebooks.

### Documentary review

Documentary sources were collected to complete our data, triangulate information and support our analysis. Documents include policy strategies related to diabetes and HIV/AIDS, but also the health sector; surveys and activity reports; training and educational tools; brochures presenting official information on actors’ priorities, strategies, and practices. During the field missions, the interviews provided opportunities to collect key resources. Throughout the study, documents were also collected online visiting the websites of key stakeholders and through internet search based on key words such as Mali, diabetes, HIV/AIDS, patient associations, patient participation, Africa and using the snowball method.^4^

### Data analysis

For the purpose of data analysis, we used an inductive and iterative approach wherein collection and analytical strategies were gradually drawn from the data itself. Recorded interviews and hand-written notes were both transcribed. After each field mission, a vertical analysis was performed, and all interviews and notes taken during observation were coded apart; a horizontal analysis was then performed for each theme, wherein we triangulated information related to each disease from different sources, including document reviews. A comparative analysis of transversal themes was finally performed between the two diseases. The analysis was progressively refined with the newly data collected in successive missions [52]. We stopped data collection when saturation was achieved, that is no new themes and no significant increase in the sophistication of analysis emerged.

Based on theories of public and collective action mentioned in the background section, a historical and cognitive approach was adopted. These theories stress the influence of past events and the importance of collective frameworks. We thus retraced the history of the fight against diabetes and HIV/AIDS and took into account the specific contexts within which patients were engaged and participatory practices were developed. We focused on actors’ perceptions and knowledge and performed a content analysis of data to explore how different actors viewed the two diseases, and how they perceived patients, their knowledge and skills, both individually and collectively. We also looked at partnerships, resources, and respective interests as these aspects influence actors’ strategies and practices.

To increase validity and reliability of our findings, data was triangulated by crossing information collected from different methods and sources at different times. In addition to the wrap-up meetings organised with local stakeholders, our findings were discussed during seminars held within the authors’ research centres and with the committee in charge of the follow-up of the first author’ work on her PhD, as well as during national and international conferences.^5^ These exchanges with other researchers were also crucial to improve and refine our analyses.

## Results

Our findings highlight that the relative openness of the Malian political system to patient participation in the fight against diabetes and HIV/AIDS is linked to political, economic, and cognitive factors. They are synthesised in Table 1 and detailed hereafter.

**Table 1:** Factors associated with participation of patient associations at policy level

Dimension	Factors	Diabetes	HIV/AIDS
Political	- extent of public authorities’ commitment	- late and slow	- early and growing
	- focus of public authorities’ commitment	- biomedical priorities	- biomedical and progressively also psychosocial priorities
	- policy-making processes put in place	- intermittent partnership between public authorities and patient associations - absence of formal frameworks for patient participation	- steadily growing partnership between public authorities and patient associations - patient participation at policy level progressively institutionalised
	- the manner patients’ role is framed by the law	- added value initially acknowledged, mostly at meso-level - patients’ role remained limited and rather passive over time	- no role for patient at the beginning - patients’ role progressively acknowledged and promoted at micro-, meso- and macro-levels
Economic	- nature of external support	- material, technical	- financial, material, technical
	- extent of external support	- low and ad hoc	- high and stable
Cognitive	- the manner the disease is perceived and framed	- biomedical perspective	- biomedical and psycho-social perspective
	- the manner patients (and their engagement) are perceived and framed by other stakeholders	- little confidence in patients - focus on patients’ engagement at micro- and meso-levels	- acknowledgment of patient expertise - added-value of patient’s engagement recognised at micro-, meso- and macro-level

### Political factors: intermittent versus steadily growing partnerships

Retracing the history of the fight against diabetes and HIV/AIDS in Mali enabled us to identify three political factors that influence the opportunities made available to patients: extent and focus of the commitment of public authorities, actual policy-making processes, and the manner in which the law frames patients’ roles.

**Fig. 1:**
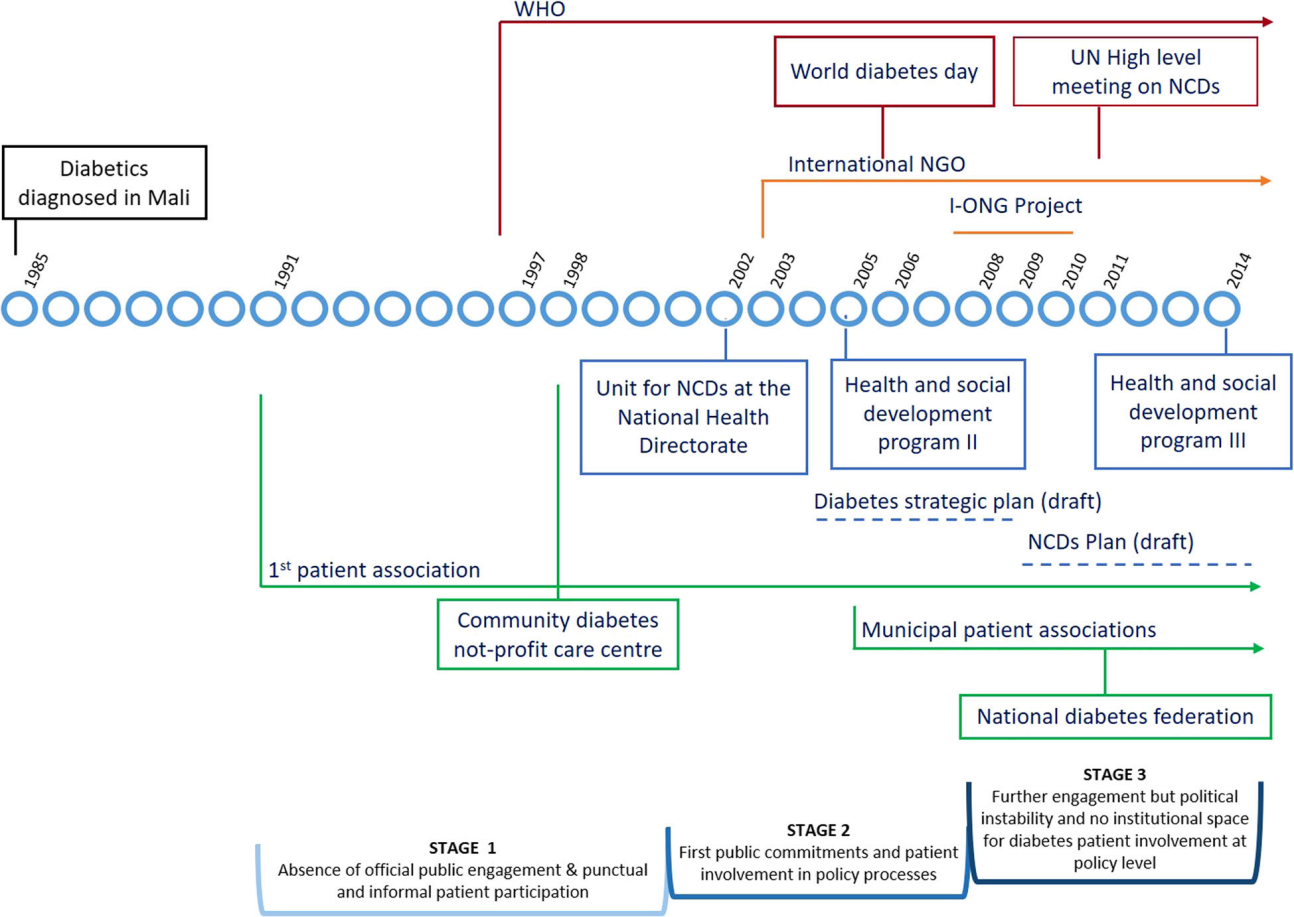
Evolution of public action on diabetes in Mali. The fight against diabetes went through three main stages that witnessed an intermittent partnership between public authorities and patient associations. Official public commitment came late and was slow, as show the few policies and authorities related to diabetes (blue lines). Malian patient associations engaged early in Bamako and rallied for a community diabetes non-profit care centre (green lines). Support from external partners was low and ad hoc. The figure stresses some international organisations and events related to diabetes at international level (red lines) and international organisations and projects that have targeted diabetes at Malian level (orange lines)

The fight against diabetes has evolved through three main stages that witnessed an intermittent partnership between public authorities and patient associations (see Fig. 1).

An absence of official public commitments during the 1990s left patients with no formal mechanisms to participate at the policy level. The need to improve diabetes care, however, pushed them to join caregivers in the first Malian diabetes association, established as early as 1991, and to rally for a community diabetes non-profit care centre, which ultimately opened its doors in 1998. At the time, patient engagement was mostly at the meso-level of service delivery, and advocacy remained focused on administrative and material needs. The status quo changed when the Malian government undertook its first ever commitment towards NCDs by setting up a specific unit in the National Health Department, and including these diseases in the five-year health and social development program 2005–2009 [53]. Thanks to these steps, patients were able to participate in multi-actor meetings organised between 2004 and 2007 to develop a strategic plan for the prevention and control of diabetes. It is to be noted that this plan was never officially adopted and remained at a draft stage. Based on its contents, the draft supported the development of associations and explicitly framed their involvement in initiatives favouring alternative financing mechanisms as well as public awareness. However, patients were not also involved in the development of educational tools nor in advocacy [54]. The situation underwent another change in late 2000s. Whereas patient movement grew with the creation of local associations in each of the six municipalities of Bamako, patient engagement declined at the political level. Once the issue of diabetes was integrated into the drafting of a comprehensive NCDs plan in 2009, the related policy-making process provided little room for patient involvement. Patient representatives revealed in 2012: “*For non-communicable diseases, it seems that some things were decided but we weren’t consulted;” “No, there was no contact at the political level*”. The draft plan for NCDs also referred to diabetes associations solely as targets of educational activities [55]. The political crisis faced by Mali since 2012 further reduced patients’ opportunities. The adoption of an official NCDs policy by Parliament was still pending towards the end of 2014. Consequently, even after the formation of a national diabetes federation in 2011, participation at the policy level remained non-institutional and informal or ad-hoc. The few initiatives held in 2014 mostly revolved around drugs supply and costs.

**Fig. 2:**
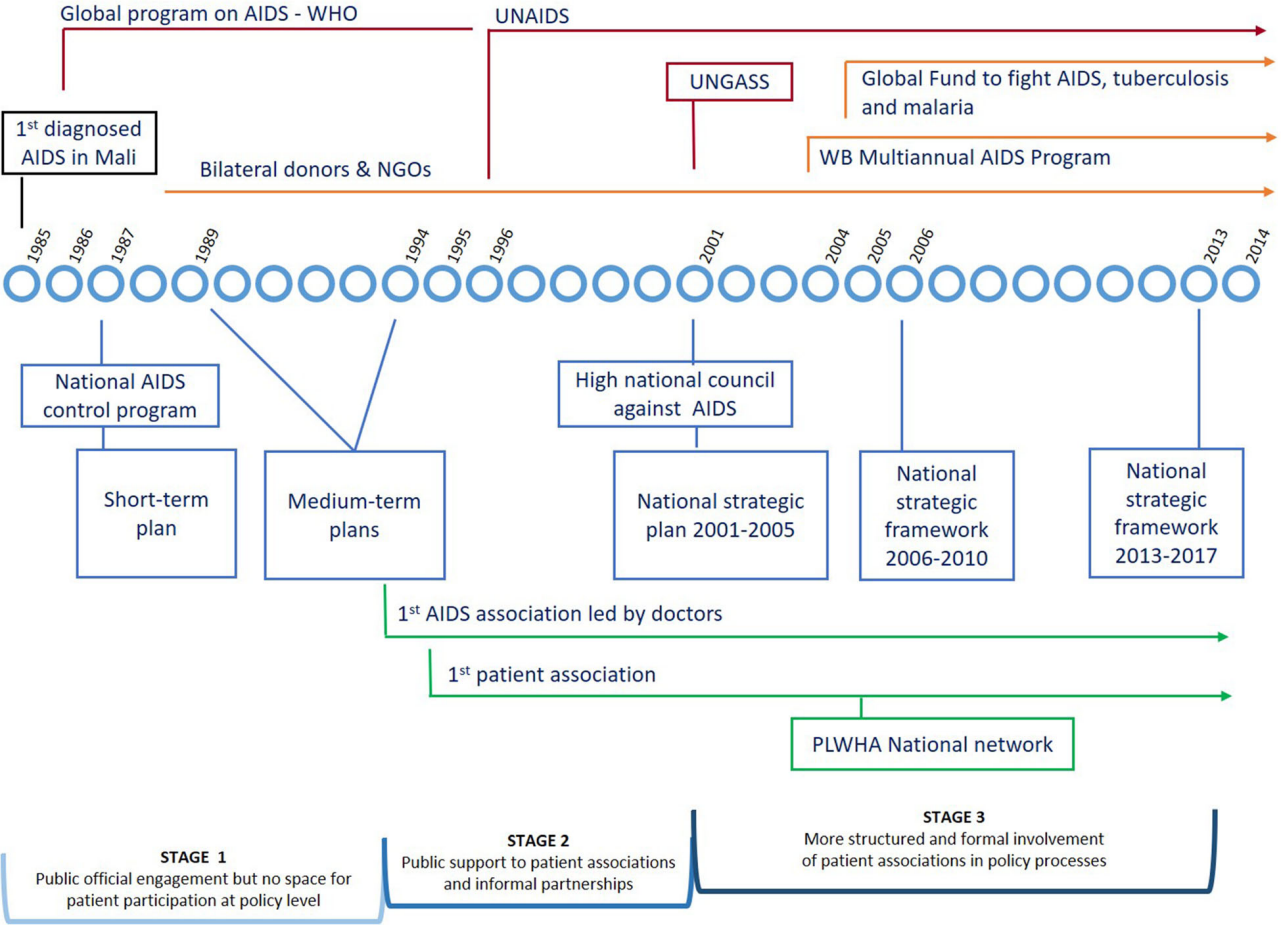
Evolution of public action on HIV/AIDS in Mali. The fight against HIV/AIDS went through three main stages that witnessed a steadily growing partnership between public authorities and patient associations. Official public commitment came early and progressively involved patient associations, as show the series of policies adopted to prevent and control HIV/AIDS (blue lines). Malian AIDS associations engaged later to complement the official strategy (green lines). External support was high and stable from the outset, including financial and/or technical support (red lines refer to organisations and events at international level; orange lines indicate organisations funding AIDS projects in Mali)

In the case of HIV/AIDS, public commitment was swifter and more dedicated: through the three stages identified in the fight against the disease, the partnership between public authorities and patients steadily grew (see Fig. 2).

A series of policies have followed the setting up of a national AIDS control program by the Ministry of Health in 1987. The first stage involved policies that were relatively technical, focused on epidemiological monitoring, prevention, and safe transfusions. Patients were mainly framed as beneficiaries, and no place was designated for patient representatives at an institutional level [56]. Things began to change from mid-1990s when associative mobilisation began to take on a formal structure. During this second stage, caregivers and patients mobilised themselves to complement the official strategy. The first association was created by doctors in 1994 to promote palliative support for patients who no longer had the possibility of hospital treatment; it created a community centre for treatment and counselling in 1996. In 1995, a patient association was born. In addition to being involved at the meso level, patients also received public support and a better standing at the policy level. This association was initially housed within the premises of the National programme and patient representatives accompanied Malian authorities at several African and international forums. The third stage has seen a more structured and formal involvement of patient associations since the 2000s. The High National Council against AIDS set up in 2001 stipulates equal representation of public, private, and civil society representatives, including patients [56]. Patient representatives have also been members of the Country Coordination Mechanism of the Global Fund (to fight AIDS, tuberculosis, and malaria); one of them served as vice-president in 2010 and president until 2013. PLWHA are today systematically consulted during the formulation of health policies. This was for example the case during the 2012 review of the national strategy on nutrition wherein PLWHA were the only patients to be consulted. Nonetheless, according to the official in charge of the revision, diabetes entailed more specific nutritional recommendations that HIV/AIDS. Overall, patient representatives seemed satisfied: *“public authorities [ …] gave a good standing to associations of people living with HIV, since, every time they wanted to do something, they did it with the associations”; “every time they talk about civil society, they mention ‘the association of people living with HIV’, it’s really clear there*!”. However, our last field study in 2014, noted relatively moderate engagement on the part of patients, arguably mirroring the downside of this “success story”. A patient representative affirmed that advocacy activities were limited other than “*perhaps to correct some shortage, but otherwise, very often, we are present at the discussions.*” A similar change was also observed at the meso level: a doctor complained of the lowered interest of newly diagnosed patients to engage in peer-support activities due to the availability of treatment, which in turn made disease management less constraining.

### Economic factors: low and ad hoc versus high and stable support

Our results demonstrate that the nature and extent of external support influences the resources and strategies of public authorities, accordingly shaping the environment for patient participation.

In the case of diabetes, Malian political commitment has closely followed that of international donors (Fig. 1). Institutional engagement emerged at a time when diabetes was starting to become a global issue. Since then, however, the only international partners of the Malian NCDs unit have been the World health organization (WHO) and an international NGO, both predominantly focused on supporting legislation and strengthening service delivery. Less financial support have been provided to the National Health Department, which currently remains extremely short-staffed. The last health and social development program (2014–2018) allocated only approximately €3 million for all NCDs [43]. This has negatively influenced the development of appropriate institutional frameworks, thereby slowing policy-making processes and reducing formal spaces and opportunities for patient participation. As it so happens, patient involvement in the policy dialogue on drug supply and costs was in fact backed by the international NGO.

In comparison, international support for HIV/AIDS has been available from the outset and has given the state a means to act. In addition to the support from the WHO and the then UNAIDS in the 1990s, financial and technical support extended by bilateral cooperation agencies was just as important. Broader programs emerged in the 2000s funded by multilateral donors, such as the World Bank or the African Development Bank, and by public-private partnerships, such as the Global Fund. This support heightened awareness amongst Malian authorities to take patients into account and to build partnerships with them. In 1995, for example, the World Health Assembly invited Member States, including Mali, to implement the principle of “greater involvement of people living with AIDS” in policy dialogue [57]. More recently, international aid conditionality has pushed through the institutional changes illustrated by the National High Council and the Global Fund Country Coordination Mechanism. Moreover, international support has provided Malian authorities with the financial resources needed to put in place inclusive policy-making processes. The fact that the health and social development program 2014–2018 allocates approximately €22 million to fight communicable diseases, in particular HIV/AIDS, malaria, and tuberculosis, [43] is a case in point.

### Cognitive factors: biomedical versus psycho-social perceptions

Our analysis reveals stark differences in how public authorities, caregivers, and donors perceive not just diabetes and HIV/AIDS, but also diabetics and PLWHA.

National strategies, for example, describe diabetes from a bio-medical perspective, stressing its epidemiological and clinical characteristics. They portray it as a public health problem “*because of its growing prevalence and high morbidity and mortality”* on a global scale ([54]: p.3), and because of “*its degenerative [ …] and acute complications.”* ([55]: p.6) The listed related problems concern the weaknesses of the health system and particularly the lack of human, material, and financial resources. Virtually unanimously, caregivers raise the same issues, as acknowledged by two doctors in 2008 and 2012 respectively: “*It is true that diabetes is a major problem in Mali, but its diagnosis is especially problematic*”; “*Although medical staff is currently well informed about diabetes, here in Bamako, there are still some doctors who cannot diagnose it correctly.”* In a similar vein, donors also appeared to be influenced by a bio-medical approach. One representative admitted in 2008*: “I would be surprised if [our agency] puts a lot more emphasis on diabetes – it is considered to be a problem for clinicians.*”

Overall, little importance is given to the psycho-social dimension of the disease. When mentioned in strategies and by stakeholders involved in the fight against diabetes, this aspect is equated with treatment and patients’ capacity for self-care. According to most documents and actors, “*the first step towards treatment [of diabetes …*] *is a change in lifestyle*” ([55]: p.6). In this regard, several social barriers to behavioural change are mentioned: economic difficulties faced by patients in adhering to a diet, cultural habits including limited physical activity, low level of education, and the subsequently poor understanding of diabetes and of its chronicity. The psychosocial impact of the disease on patients was scarcely touched upon by the actors we met. Only one NGO representative freely referred to its psychological consequences in 2010: “*It’s not that easy to see, but during [a particular] activity, I noticed that diabetic people are introspective, they keep to themselves [ … They] do not even like to admit that they are diabetic”*. However, the interviewee associated patients’ isolation to their poor understanding of the disease. In fact, diabetes is rarely linked to social discrimination. This is most evident when speaking with actors less concerned by diabetes, such as those involved in the fight against AIDS, who unanimously regarded diabetes as being socially better accepted. Like other international partners, an NGO representative stated in 2010: “*People easily say that they are diabetic; they say that they can’t drink this or that because of the sugar*”.

When examining actors’ perception of diabetics, we note that little confidence is usually given to patients’ abilities to care for themselves or their peers. With respect to individual treatment, a caregiver conceded in 2012: “*It’s disastrous, [ …] no diabetic in Mali sticks to their diet; even doctors who are diabetic do not stick to their diets*”. In the same year, another caregiver referred to peer-educator activities launched by patient associations to make his point: “*Since there was no doctor [ …] to give medicines, some people saw no interest in coming to sit with peers or others who are not medically trained, who have the same disease and who might be willing to give information on it. They thought it was a waste of time*”.

The dominance of a bio-medical perspective as well as little trust in patient ability and expertise therefore concern patients’ potential role equally at the policy level. One of the first drafts of the diabetes strategic plan highlighted the “strong [early] mobilisation of diabetic patients”, but remained focused on their contribution at the meso-level of service delivery ([54]: p.4). In 2010, written advocacy by municipal associations for diabetes subsidies and free treatment remained unanswered. A national official maintained: “*These are really one-off actions, [ …] temporary: we go there, the patient writes, we go to give him medicines, to give him money and then it’s over. Whereas, for us, that is not what is needed – we need to be able to develop the system*”. Two years later however, the same official offered a renewed take on patient mobilisation, deeming the federation “*a good thing [ …] to help identify the problems, to see how we can deal with them together.*”

Specific to HIV/AIDS, strategies also address bio-medical concerns of the disease related to its epidemiological and clinical dimensions. Yet, unlike diabetes, interviews and policy documents systematically mention its social and psychological impact as well. In 2010, two caregivers remembered how, during the 1990s, *“HIV/AIDS was a fatal disease – people were afraid*”; it was “*the disease without medicines*” doctors did not dare announce to their patients. In this context, attention gradually shifted to the quality of life of PLWHA, the need to support them, and the need to respect their rights and dignity. Even after the introduction of antiretroviral therapy and its free supply in 2001, the psychosocial dimension remained a key issue due to discrimination and stigmatisation of PLWHA. The 2001 strategic plan presented AIDS as “*a priority public health problem [because of the] amplitude taken by the epidemic [ …] and its negative impact on the population’s well-being.*” ([56]: p.64) Among others, it identified the following problems: “*the absence of psychosocial care, stigmatisation, breach of confidentiality or deprivation of rights.*” ([56]: p.28) In 2010, this vision still permeated actors’ views. A donor representative professed: “*with AIDS, there’s stigmatisation and discrimination [ …] – that’s not the same thing with diabetes*”.

The social context of HIV/AIDS, coupled with the initial scientific uncertainty, opened opportunities for patient engagement in care and peer-to-peer support as well as to acknowledge their expertise and added-value. A doctor confirmed in 2010: “*This aspect of psychosocial accompaniment and support was something truly important at the start because it was given by people who were infected and affected, by volunteers; [ …] Patients have experience that they can transfer, [ …] that they can testify to. They too have gone through trials and tribulations, so all these are points that no one has taught me in medical school!”*

Patients’ expertise and ability to engage was equally recognised when talking about their mobilisation at the policy level. According to another doctor: “*Our health and political authorities were always accompanied by a very strong civil society*” (2010). Explaining the rationale behind involving PLWHA during the revision process of the nutrition strategy in 2012, the official in charge of it asserted: *“[they] know more about their disease, [ …] about nutrition, they know what they should or shouldn’t consume”*.

## Discussion

Patient participation is a social construct that depends on multiple dimensions. These aspects influence the social standing of patients, as well as the role they take (or decide not to take) [21, 58, 59]. This article explored the factors associated with the opportunities given to chronic patients in the fight against diabetes and HIV/AIDS in Mali, notably at the policy level. We focused on the openness of the institutionalised political system and looked at strategies and discourses of actors other than patients involved in policy-making (national authorities, caregivers, and donors). Based on theories of public and collective action, we investigated not only public policies and spaces formally related to diabetes and HIV/AIDS, but we explored more broadly all actions engaged by stakeholders and the frameworks they support about these two public health issues [60].

We adopted a historical and comparative approach, which showed how the patient standing has changed over time and according to the disease at stake. Hence, at different stages of the fight against diabetes and HIV/AIDS in Mali, patients either *appeared* or *disappeared* from policy contents and policy-making processes. And today, patient participation depends on the nature of their pathology: what is announced and built in favour of participation of PLWHA does not necessarily apply to other patients. Progress made in the fight against HIV/AIDS remains non-universal and has little changed practices in other health-related fields. As Saout pointed out, changes obtained for HIV/AIDS remain “fragile achievements” [61]. Our results thus challenge the image of an “active patient” conveyed by strategies related to chronic diseases. They reveal how *chronic* patients are more so *intermittent* partners for policy-makers and partnerships with public authorities are not straightforward, nor stable. Baszanger has come to the same conclusion for patient participation at the meso-level, with patients supposedly at the centre of a healthcare system when, in actuality, they “are just passing through” ([62]: p.92). Several intertwining factors related to political, economic, and cognitive dimensions may explain the intermittent nature of this partnership.

### Relationships between states, patients and donors at stake

Specific to political factors, we observed that the focus and extent of the commitment of public authorities to diabetes and HIV/AIDS influences opportunities for patient engagement and creates room for their effective involvement. In the case of diabetes, patients engaged early to fill the void left by national authorities and international donors. However, their initial status as key partners progressively declined, partly because of the absence of formal frameworks for policy dialogue. Conversely, in the case of HIV/AIDS, patients involved themselves to complement prior public commitments, which in turn accompanied and potentially framed the growing patient engagement. This role of the state has been found in other contexts, such as participation of AIDS associations in research made in France [34]. State-society relationship is now acknowledged to be a key governance component [63], and understanding these relational dynamics is crucial in building participatory strategies.

Links made with international actors are also at stake. Literature on public policy in Africa highlights the key role played by international partners. Lavigne Delville speaks about “assumed coproduction of public policies between state and donors” ([64]: p.16), and Eboko refers to “public co-action” [28]. Our study shows that the economic support provided by international donors has shaped institutional opportunities for participation. Depending on the nature and extent of their assistance, donors have offered strategic models for public strategies and had an impact on the resources available to implement them. With diabetes, the limited and largely sporadic international partnerships have strongly constrained public resources and commitments. We have elsewhere revealed how a lack of resources resulted in Malian authorities being trapped in a vicious circle. Their inability to conduct national and systematic epidemiological surveillance on diabetes left them without adequate data to prioritise and rally with donors, who in turn had one more argument not to support the fight against diabetes [65]. With respect to HIV/AIDS, other studies emphasise that public support to AIDS associations was a clear strategy to align Mali with the national programmes of other African countries [36]. The fact that creating multi-actor institutions was a formal condition to benefitting from World Bank funding is also a case in point [49].

### The importance of cognitive frameworks related to the disease

We argue here that the cognitive dimension is also at stake, notably in relation to the way diseases are perceived and framed by actors, such as policy-makers, caregivers, and donors. Effectively, the types of shared diagnostic frameworks impact the solutions and expected contributions from patients, whether individual or collective. In the case of diabetes, its perception as a primarily bio-medical problem led policy-makers to expect solutions “*from above”*: from clinicians providing relevant policy recommendations and quality care; from public institutions developing effective strategies; and from donors supporting technical and financial resources. Moreover, the view that diabetes is a chronic, non-communicable disease that can be easily managed over the long term confined patient responsibility to their individual care. As a result, fewer solutions were expected from their collective engagement. A similar biomedical approach has also dominated the first strategies concerning HIV/AIDS in Mali, much like in other African contexts [66]. And yet, anthropological studies reveal that Malian public authorities initially ignored the relevance of the disease [36]. This situation left little room to involve patients at the policy level during the first years of the epidemic. It was due to the stigma faced by patients that the psycho-social dimension of HIV/AIDS was increasingly embraced by both national authorities and caregivers who gradually sought solutions “from the bottom-up” involving people both infected and affected by the disease.

It is important to note that the manner in which actors perceive a disease also depends on the scientific and medical knowledge available on it. Scientific knowledge on diabetes has been around for a long time, particularly in Northern countries where the disease is routinely treated, and its chronicity experienced by patients. We maintain that this routine and chronic experience currently affects donors’ view of the disease, and partially explains why they tend to underestimate its consequences, accordingly accentuating the bio-medical dimension more than the psycho-social one. This view contrasts with findings from anthropological studies conducted in Mali [67, 68], and in contexts as diverse as South-Africa, Reunion Island or Australia [69–71]. Those studies reveal the emotional suffering of diabetic patients. In the case of Mali for example, they have come to see diabetes as “worse” than HIV/AIDS, for which treatment today is free of charge [68]. The risk that the notion of chronicity may obscure “the protracted uncertainty and precarious life conditions experienced” by patients and may result in reduced international support has been recently underscored by articles alerting against the ‘end of AIDS’ discourse ([72]: p.992, [73]).

### The issue of patients’ legitimacy

Amongst the cognitive factors associated with the building of patient participation, our analysis also underlines the manner in which patients themselves are perceived by policy-makers, caregivers, and donors. As demonstrated by our results, in the case of diabetes, most testimonies depict a relatively *“undisciplined chronic patient”*, who does not adhere to his diet nor understand the chronicity of the disease. This perception negatively influences other actors’ recognition of diabetics’ experiential knowledge, and correspondingly affects their legitimacy to participate in decision-making. Conversely, it is the recognition of PLWHA expertise and added value at the meso-level, within peer-support activities for example, that boosts their recognition as credible partners also at the policy level. We assert that the role assigned to patients at the micro- and meso-level, based on their long-term experience with self- and group care, has an impact on framing patients’ legitimacy at the macro-level.

As for the diseases themselves, actors’ perception of patients is also influenced by the scientific context. Where medical knowledge is available and validated by the scientific and political community, much like for NCDs, patients encounter greater difficulties in having their knowledge recognised, as witnessed in France by cancer patients associations [74]. Anthropological analyses on diabetes knowledge in Mali equally reflects this trend [68]. Conversely, in the case of HIV/AIDS, treatment was initially unknown and patient involvement emerged in the context of a “therapeutic and social slump” [34]. Studies conducted in different contexts, such as USA or France, show how the absence of this scientific knowledge enabled patients to have a standing in scientific research, to question medical authority, and to express their voice [33, 34]. It is important to note that the status quo is not fixed and may change over time, even for HIV/AIDS. Our results underscore the risk of patients’ disengagement from collective initiatives when faced with well-known and available treatments. At the beginning of the 2000s, the “*return of the singularity of patients*” had already been called attention to on account of the availability of antiretroviral drugs and the normalisation of HIV/AIDS in Northern countries [75].

### Limitations

Our fieldwork spanned from 2008 to 2014. This allowed us to observe changes in the way policies and actors evolved over time. In the case of diabetes, the weak international commitment and the scarcity of resources available to both public and associative actors have made change very slow, with the majority of our 2008 findings still holding true at the end of our study. With respect to HIV/AIDS, our historical approach also allowed to acknowledge the initial difficulties faced by patient associations at the outset of the fight against the disease and to tone down the satisfaction that stakeholders show today in relation to current policy involvement of PLWHA. The initial difficulties were testified by actors we met and which were amongst the first Malian activists, as well as by literature on AIDS mobilisation in Mali during the 1990s [36, 76]. Also, limits of participatory mechanisms have been widely demonstrated, including in the context of the Global Fund [77].

As stated in the method section, a second round of interviews initially planned with actors engaged in the fight against HIV/AIDS could not take place. Data was triangulated and completed by referring to documents, press articles and related literature.

Our last mission in 2014 detected a new – contrasting - trend in the case of both diseases: diabetes patients received a heightened role at the policy level, in part related to their merging into a national federation. In contrast, there was relatively moderate engagement of PLWHA both at policy and care levels, linked to the normalisation of their policy involvement and treatment. Further research on what appears to be a fourth stage in the fight against diabetes and HIV/AIDS would be useful to attest to the changes in patient participation over time and corroborate our findings.

Finally, our work focused on Bamako, since central public authorities, specialised treatment centres, and main national and international stakeholders are based there. However, this context differs from other regions in Mali, particularly rural areas, wherein other dynamics would arguably explain patient participation.

## Conclusions

Drawing upon examples of the fight against diabetes and HIV/AIDS in Mali, our study shows how chronic patients are *intermittent partners* for policy-makers. Despite the image of *chronic active patients* conveyed by national and international public health strategies, their participation is not straightforward. Rather, political, economic, and cognitive factors underpin the presence (or absence) of political opportunities that enable patient participation. It is worth noting that chronicity of the disease appears to play an ambiguous role in the shaping of these factors. We question whether the view of chronicity as being manageable, stable, or relatively normal has so far affected the participation of diabetes patients, and whether it currently limits PLWHA participation due to the normalisation of HIV/AIDS. All in all, it is the nature of the disease – its biomedical and psychosocial characteristics – and the scientific knowledge about it that play a crucial role in influencing the perceptions and public and economic commitments of stakeholders involved.

## Additional file

Additional file 1: Interviews related to public action around diabetes. (DOCX 27 kb)

Additional file 2: Interviews related to public action around HIV/AIDS. (DOCX 20 kb)

Additional file 3: Non-participant observation related to diabetes. (DOCX 14 kb)

Additional file 4: Non-participant observation related to HIV/AIDS. (DOCX 14 kb)

## Data Availability

The data that support the findings of this study are available from the corresponding author upon reasonable request.

## Abbreviations

AIDS: Acquired immunodeficiency syndrome
HIV: Human immunodeficiency virus
NCD: Non-communicable diseases
NGO: Non-governmental organisation
PLWHA: People living with HIV/AIDS
UNAIDS: Joint United Nations Programme on HIV/AIDS
WHO: World Health Organization

## Footnotes

1 In total, 10 people could not be interviewed. Most of them were not available during the field mission period; some donors refused given their little involvement in the health sector; only 1 appointment was explicitly cancelled, without explanation. [see Additional file 1].

2 Representatives were recruited due to their involvement at the policy level. Ordinary patients were interviewed by the second author as part of another component of our research; findings were developed in articles we refer to in the discussion.

3 An additional mission had been planned for May 2012 but could not be carried out due to political and security issues. In total, 5 people could not be interviewed due to their unavailability during the field mission periods [see Additional file 2].

4 Selection of new sources of information based on the references within relevant documents.

5 Amongst others: Martini J, Tijou Traoré A, Mahieu C. “Does chronicity necessarily lead to patient policy participation? Diabetes & HIV/AIDS cases in Mali », 9th European Public Health Conference, 9–12 November 2016, Vienna.
