# Supplementary material for: Chronic patient as intermittent partner for policy-makers: the case of patient participation in the fight against diabetes and HIV/AIDS in Mali
Source: BMC Public Health. 2019 Aug 28;19:1179. doi: 10.1186/s12889-019-7453-2 (PMC6712700; doi:10.1186/s12889-019-7453-2)
Supplement: Supplementary file 1 — Interviews related to public action around diabetes. (DOCX 27 kb) [file 12889_2019_7453_MOESM1_ESM.docx]

# Additional file 1 - Interviews related to public action around diabetes

Each interviewee is identified by a case code. The case code is composed as follow: the acronym related to the category of stakeholders represented by the interviewee + the first letter of the disease (e.g. D for diabetes) + a number per person interviewed for each category. For each person, we specified his/her function within the organisation, and a unique line is usually used per each person: his/her case code is repeated into multiple columns when the person was interviewed during several field missions. Sometimes, two different codes are used for the same line: this means that two different persons were interviewed over time for the same function within the organisation, given the turnover of the staff; an asterisk (*) distinguishes these case codes.

The number of actors interviewed is indicated in brackets in the first column for all stakeholders and per each category of stakeholder (N). Most interviews were conducted individually; some were in groups of two or three people depending on the interviewees’ availability. When an interviewee participated to a group interview, his/her code his completed by the letter G (^G^) + a number to distinguish the related group. The total number of interviews conducted either individually or in group is given at the end of the table for each field mission. For the mission held in 2014, actors who participated to final wrap-meetings are distinguished by the symbol ^■^.

Case codes are underlined when tape recording was refused by the interviewee or not possible.

| **Stakeholders**  **(N=55)** | **Organisation** | **Function** | **April-July 2008**  **(12 weeks)** | **May-June 2010**  **(3 weeks)** | **February-March 2012**  **(2 weeks)** | **November**  **2014**  **(1 weeks)** |
| --- | --- | --- | --- | --- | --- | --- |
| **Public administration (PA)**  **N=9** | National health directorate | Head of unit for NCDs | PA-D1 | PA-D1 | PA-D1 | PA-D1 |
|  |  | Head of division for the prevention and control of the disease | PA-D2* |  | PA-D6* |  |
|  |  | Policy officer on nutrition |  |  | PA-D7 |  |
|  | Ministry of health | Head of department of traditional medicine | PA-D3 |  |  |  |
|  | Organisation in charge of drug supply | Director of distribution department | PA-D4 |  |  |  |
|  |  | Head of division sale and delivery | PA-D5 |  |  |  |
|  |  | Deputy director |  |  | PA-D8 |  |
|  |  | Chief-executive |  |  | PA-D9 |  |
| **Caregivers (C)**  **N=5** | Community centre | Doctor | C-D1 | C-D1 |  | C-D1^■^ |
|  |  | Doctor | C-D2 | C-D2 | C-D2 |  |
|  |  | Nurse |  | C-D4 |  |  |
|  |  | Doctor |  |  | C-D5 | C-D5^■^ |
|  | Hospital | Doctor | C-D3 |  |  |  |
| **Patient associations (P)**  **N=8** | Association A | Spokesperson | P-D1 |  |  |  |
|  |  | Spokesperson |  |  | P-D4 | P-D4^■^ |
|  | Association B | Spokesperson | P-D2 |  | P-D2 |  |
|  | Association C | Spokesperson |  | P-D3 | P-D3 ^G 1^ | P-D3 |
|  |  | Treasurer |  |  | P-D5 ^G 1^ |  |
|  | Association D | Spokesperson |  |  | P-D6 | P-D6 |
|  | Association E | Spokesperson |  |  | P-D7 |  |
|  | Association F | Spokesperson |  |  | P-D8 |  |
| **NGOs**  **(NG)**  **N=16** | Local NGO | Programme manager | NG-D1 |  |  |  |
|  |  | Deputy director |  | NG-D09 ^G 2^ |  |  |
|  |  | Project manager |  | NG-D10 ^G 2^ |  |  |
|  | INGO A | Programme manager | NG-D2 |  |  |  |
|  | INGO B | Project manager | NG-D3* | NG-D11* |  |  |
|  |  | Task officer | NG-D4* | NG-D12* |  |  |
|  |  | Task officer | NG-D5 |  |  |  |
|  | INGO C | Chief executive officer | NG-D6 | NG-D6 |  | NG-D6 |
|  |  | Project manager | NG-D7* |  | NG-D13* ^G3^ |  |
|  |  | Project manager | NG-D8* |  | NG-D14* ^G3^ |  |
|  |  | Assistant project manager |  |  | NG-D15 ^G 3^ |  |
|  |  | Deputy director |  |  | NG-D16 |  |
| **Donors (health and/or social programme) (D)**  **N=17** | African development bank | Expert | D-D1 |  |  |  |
|  | Belgium | Technical assistant | D-D2 |  |  |  |
|  | Canada | Program manager | D-D3 |  |  |  |
|  | European Union | Program manager | D-D4 |  |  |  |
|  | FAO | Program manager | D-D5 |  |  |  |
|  | France | Program manager | D-D6* |  | D-D17* |  |
|  |  | Regional advisor | D-D7 |  |  |  |
|  |  | Technical advisor |  | D-D15 |  |  |
|  | The Netherlands | Advisor | D-D8 |  |  |  |
|  | Switzerland | Programme manager | D-D9 |  |  |  |
|  |  | Deputy director |  | D-D16 |  |  |
|  | UNICEF | Expert | D10 |  |  |  |
|  | USA | Advisor | D-D11 |  |  |  |
|  | WHO | Head of unit | D-D12 | D-D12 | D-D12 |  |
|  | World Bank | Expert | D-D13 |  |  |  |
|  | World Food Programme | Programme manager | D-D14 |  |  |  |
| ***Number of interviews conducted individually*** | | | ***32*** | ***11*** | ***15*** | ***4*** |
| ***Number of interviews conducted in group*** | | | ***0*** | ***1*** | ***2*** | ***1***^■^ |

In total, 10 people could not be interviewed. Details are given in the table below.

| **Category** | **Mission 2008** | **Mission 2010** | **Mission 2012** | **Mission 2014** |
| --- | --- | --- | --- | --- |
| **Public administration (PA)** | Nutrition division at National health directorate 🡪 head of division not available | Division for the prevention and control of the disease at National health directorate 🡪 head of division not available | -- | -- |
|  | Pharmacy and medicines directorate 🡪 deputy director not available | -- |  |  |
| **Caregivers (C)** | -- | -- | -- | Community centre 🡪 Doctor not available |
| **Patient associations (P)** | -- | -- | Patient association of one municipality (out of 6) 🡪 representative could not be reached | Youth diabetes associations 🡪 appointment with representative cancelled |
| **NGOs (N)** | -- | -- | International NGO 🡪 Chief executive officer not available | -- |
| **Donors (health and/or social programme) (D)** | Belgian Embassy 🡪 Program officer on leave | -- | -- | -- |
|  | Germany 🡪 no health program | -- |  |  |
|  | UNDP 🡪 no health program | -- |  |  |
| **TOTAL** | 5 | 1 | 2 | 2 |
