# Supplementary material for: Chronic patient as intermittent partner for policy-makers: the case of patient participation in the fight against diabetes and HIV/AIDS in Mali
Source: BMC Public Health. 2019 Aug 28;19:1179. doi: 10.1186/s12889-019-7453-2 (PMC6712700; doi:10.1186/s12889-019-7453-2)
Supplement: Supplementary file 2 — Interviews related to public action around HIV/AIDS. (DOCX 20 kb) [file 12889_2019_7453_MOESM2_ESM.docx]

# Additional file 2 - Interviews related to public action around HIV/AIDS

Each interviewee is identified by a case code. The case code is composed as follow: the acronym related to the category of stakeholders represented by the interviewee + the first letter of the disease (e.g. A for HIV/AIDS) + a number per person interviewed for each category. For each person, we specified his/her function within the organisation, and a unique line is usually used per each person: his/her case code is repeated into both columns when the person was interviewed during both field missions.

The number of actors interviewed is indicated in brackets in the first column for all stakeholders and per each category of stakeholder (N). Most interviews were conducted individually; some were in groups of two or three people depending on the interviewees’ availability. When an interviewee participated to a group interview, his/her code his completed by the letter G (^G^) + a number to distinguish the related group. The total number of interviews conducted either individually or in group is given at the end of the table for each field mission. For the mission held in 2014, actors who participated to final wrap-meetings are distinguished by the symbol ^■^.

Case codes are underlined when tape recording was refused by the interviewee or not possible.

| **Stakeholders**  **(N=27)** | **Organisation** | **Function** | **May-June 2010** | **November**  **2014** |
| --- | --- | --- | --- | --- |
| **Public administration**  **(PA)**  **N=6** | Executive Secretariat of the National High Council for the Fight against AIDS | Responsible for private sector | A-A1 |  |
|  |  | Head of department mobilisation of national response | A-A2 |  |
|  | Ministry of health, coordination unit of the sector-committee against AIDS | Responsible of infant care | A-A3^G1^ |  |
|  |  | Responsible of PMTCT | A-A4 ^G 1^ |  |
|  |  | Responsible of care | A-A5 |  |
|  | Ministry of social development, solidarity and old people, coordination unit of the sector-committee against AIDS | Coordinator | A-A6 |  |
| **Caregivers**  **(C)**  **N=3** | Community care-centre | Doctor | C-A1 |  |
|  |  | Doctor |  | C-A2^■^ |
|  |  | Doctor |  | C-A3^■^ |
| **Patient associations**  **(P)**  **N=4** | Network of patient associations | Spokesperson | P-A1 ^G 2^ | P-A1 |
|  |  | Spokesperson | P-A2 ^G 2^ |  |
|  |  | Spokesperson | P-A3 ^G 2^ |  |
|  |  | Spokesperson |  | P-A4^■^ |
| **NGOs**  **(NG)**  **N=10** | Local NGO A | Director | NG-A1 |  |
|  |  | Project assistant |  | NG-A9^■^ |
|  |  | Coordinator |  | NG-A10^■^ |
|  | Local NGO B | Deputy director | NG-A2 ^G 3^ |  |
|  |  | Project manager | NG-A3 ^G 3^ |  |
|  |  | Programme manager | NG-A4 ^G 4^ |  |
|  |  | Team leader | NG-A5 ^G 4^ |  |
|  | INGO A | Project manager | NG-A6 |  |
|  | INGO B | Coordinator | NG-A7 ^G 5^ |  |
|  |  | Accounting manager | NG-A8 ^G 5^ |  |
| **Donors  (AIDS program)**  **(D)**  **N=4** | UNAIDS | Director | D-A1 |  |
|  |  | Advisor | D-A2 |  |
|  | UNICEF | Project manager | D-A3 |  |
|  | WHO | Programme manager | D-A4 |  |
| ***Number of interviews conducted individually*** | | | **11** | **1** |
| ***Number of interviews conducted in group*** | | | **5** | **1^■^** |

In total, 5 people could not be interviewed given their unavailability during the field mission period. Details are given in the table below.

| **Category** | **Mission 2010** | **Mission 2014** |
| --- | --- | --- |
| **Public administration (PA)** | Ministry of health, coordination unit of the sector-committee against AIDS 🡪 Director not available | -- |
| **Caregivers (C)** | -- | Community center 🡪 Doctor not available (replaced by two colleagues at the wrap-up meeting) |
| **Patient associations (P)** | Women associations 🡪 representatives not available | -- |
| **NGOs (NG)** | -- | Local NGO 🡪 Director not available (replaced by two program officers at the wrap-up meeting) |
| **Donors (health and/or social programme) (D)** | WB 🡪 program officer not available | -- |
|  | USAID 🡪 program officer not available |  |
| **TOTAL** | 4 | 2 |
