# Supplementary material for: Chronic patient as intermittent partner for policy-makers: the case of patient participation in the fight against diabetes and HIV/AIDS in Mali
Source: BMC Public Health. 2019 Aug 28;19:1179. doi: 10.1186/s12889-019-7453-2 (PMC6712700; doi:10.1186/s12889-019-7453-2)
Supplement: Supplementary file 3 — Non-participant observation related to diabetes. (DOCX 14 kb) [file 12889_2019_7453_MOESM3_ESM.docx]

# Additional file 3 - Non-participant observation related to diabetes

| **Description** | **Period** | **Days** |
| --- | --- | --- |
| Training on diabetes care for doctors and nurses of decentralised care centres, organised by the international NGO, in partnership with local health professionals and the National Health Department | May 2008 | 4 |
| Training on diabetes care for doctors and nurses of decentralised care centres, organised by the international NGO, in partnership with local health professionals and the National Health Department | May 2010 | 4 |
| Visit to the diabetes unit at a municipal health facility | March 2012 | 0.5 |
| Closing ceremony of World Diabetes Day organised by a patient association | November 2014 | 0.5 |
