# Supplementary material for: Chronic patient as intermittent partner for policy-makers: the case of patient participation in the fight against diabetes and HIV/AIDS in Mali
Source: BMC Public Health. 2019 Aug 28;19:1179. doi: 10.1186/s12889-019-7453-2 (PMC6712700; doi:10.1186/s12889-019-7453-2)
Supplement: Supplementary file 4 — Non-participant observation related to HIV/AIDS. (DOCX 14 kb) [file 12889_2019_7453_MOESM4_ESM.docx]

# Additional file 4 - Non-participant observation related to HIV/AIDS

| **Description** | **Period** | **Days** |
| --- | --- | --- |
| Awareness campaign held within a community centre | May 2010 | 1 |
| Meeting held at WHO headquarter between donors funding HIV/AIDS, national authorities and local civil society | June 2010 | 0,5 |
